# 2167 Beyond diagnosis: Using ultrasound to affect tumor vasculature for hepatocellular carcinoma (HCC) therapy

**DOI:** 10.1017/cts.2018.50

**Published:** 2018-11-21

**Authors:** Julia D’Souza, Laith Sultan, Sean Carlin, Terence Gade, Stephen Hunt, Chandra Sehgal

**Affiliations:** School of Medicine, University of Pennsylvania

## Abstract

OBJECTIVES/SPECIFIC AIMS: Preliminary animal studies showed that low-intensity ultrasound (US) coincident with intravascularly administered microbubbles locally disrupts tumor vasculature. This study translates the novel therapy of antivascular ultrasound (AVUS) into an autochthonous model of hepatocellular carcinoma (HCC). The differential effects produced by AVUS at low and high doses are evaluated. METHODS/STUDY POPULATION: HCC was induced in 12 Wistar rats by ingestion of 0.01% diethylnitrosamine in drinking water for 12 weeks. Rats received AVUS treatment at low and high doses. Low dose group (n=6) received 1 W/cm^2^ US for 1 minute with 0.2 mL microbubbles injected IV. High dose group (n=6) received 2 W/cm^2^ for 2 minute with 0.7 mL microbubbles IV. Perfusion was measured before and after AVUS with contrast-enhanced ultrasound (CE-US) and power Doppler (PD-US). Peak enhancement (PE) and perfusion index (PI) were measured from each US mode. Histology after sacrifice or natural death was compared to pre/post US. Analysis of H&E and trichrome sections was evaluated for percent area of hemorrhage and findings of tissue injury and repair including inflammation, necrosis, and fibrosis. RESULTS/ANTICIPATED RESULTS: After high dose AVUS, PE, and PI of CE-US decreased from baseline by an average of 33.3% and 29.7%, respectively. Histology showed extensive tissue injury (hemorrhage, necrosis, fibrosis) in 58% of tumor cross-sectional area. Conversely, low dose AVUS increased PE and PI of CE-US by an average of 39.3% and 67.8%, respectively. Histology showed smaller areas of microhemorrhage Versus large pools of hemorrhage (only 17% area). PD-US changes were similar to CE-US. DISCUSSION/SIGNIFICANCE OF IMPACT: In summary, the opposing effects of AVUS observed at 2 doses allows for multiple roles in tumor therapy. Enhanced perfusion at a low dose may improve drug delivery or radiation therapy. Whereas, vascular disruption at high doses of AVUS may allow noninvasive ischemic therapy. Furthermore, AVUS is ripe for translation given the use its component parts clinically: low-intensity long-tone burst for physiotherapy and microbubbles as an US contrast agent. Thus, AVUS should be evaluated for translation of its differential effects into noninvasive therapies for HCC and other tumors.

